# Differences between immigrant and non-immigrant groups in the use of primary medical care; a systematic review

**DOI:** 10.1186/1472-6963-9-76

**Published:** 2009-05-11

**Authors:** Ellen Uiters, Walter Devillé, Marleen Foets, Peter Spreeuwenberg, Peter P Groenewegen

**Affiliations:** 1 NIVEL, Netherlands Institute for Health Services Research, Utrecht, The Netherlands; 2 RIVM, National Institute for Public Health and the Environment, Bilthoven, The Netherlands; 3 Institute of Health Policy and Management, Erasmus Medical Center, Rotterdam, The Netherlands

## Abstract

**Background:**

Studies on differences between immigrant and non-immigrant groups in health care utilization vary with respect to the extent and direction of differences in use. Therefore, our study aimed to provide a systematic overview of the existing research on differences in primary care utilization between immigrant groups and the majority population.

**Methods:**

For this review PubMed, PsycInfo, Cinahl, Sociofile, Web of Science and Current Contents were consulted. Study selection and quality assessment was performed using a predefined protocol by 2 reviewers independently of each other. Only original, quantitative, peer-reviewed papers were taken into account. To account for this hierarchical structure, logistic multilevel analyses were performed to examine the extent to which differences are found across countries and immigrant groups. Differences in primary care use were related to study characteristics, strength of the primary care system and methodological quality.

**Results:**

A total of 37 studies from 7 countries met all inclusion criteria. Remarkably, studies performed within the US more often reported a significant lower use among immigrant groups as compared to the majority population than the other countries. As studies scored higher on methodological quality, the likelihood of reporting significant differences increased. Adjustment for health status and use of culture-/language-adjusted procedures during the data collection were negatively related to reporting significant differences in the studies.

**Conclusion:**

Our review underlined the need for careful design in studies of differences in health care use between immigrant groups and the majority population. The results from studies concerning differences between immigrant and the majority population in primary health care use performed within the US might be interpreted as a reflection of a weaker primary care system in the US compared to Europe and Canada.

## Background

Equity in access to health care services has been a major concern among many western countries in the past decades. Equity refers to the extent to which access is determined by 'medical need' as proxied by health status as opposed to socio-economic factors such ethnicity, income and insurance status [1]. Research addressing this issue often focuses on the variation in health care use according to social categories such as gender, immigrant status and socio-economic position. With respect to immigrant status, a substantial body of literature has documented differences between immigrant groups and non-immigrant groups in health care utilization [2-13]. Nevertheless, these studies do not always agree about the extent and direction of differences in health care use or the relative importance of the explaining variables, which makes it difficult to draw general conclusions.

One possible way of drawing conclusions on the basis of a body of research is to perform a systematic review. Reviewing the international literature provides a means to study differences in health care utilization between immigrant and non-immigrant groups from a broad perspective. Even though countries have different immigration histories (and hence different immigrant groups) and dissimilar health care systems, international literature concerning differences between immigrant and non-immigrant groups in use of health care is relevant in revealing to what extent (determinants of) these differences are universal or country-specific. Insight into the role of different determinants of health care utilization allows us to establish to what degree differences in utilization reflect differences in health care needs and in accessibility of health care systems.

This paper assesses differences between immigrant and non-immigrant groups in health care use in a systematic way. The focus will be on the use of primary medical care. Health care systems differ widely between countries in terms of reimbursement system, the gate-keeping role of the family physician and the size of practices (small doctor's offices, large health care centres). However, primary care in general serves as an entry point to the complex health care system and provides a link to more specialized care. Strong primary care systems are associated with a health-enhancing impact [14]. Given this relationship between primary care and health status it is important to identify disparities in the use of this type of care [15-17]. Part of a systematic review is the assessment of the methodological quality of the studies. This way more insight is provided in the association between study quality and study results. The following research questions were formulated:

1 Are differences between immigrant and non-immigrant groups in the use of primary medical care systematically found across countries and immigrant groups?

2 To what extent is the significance of differences between immigrant and non-immigrant groups in primary medical care use related to study characteristics, strength of the primary care system and the methodological study quality?

## Methods

The review has been performed by using a predefined protocol in which the following criteria for inclusion were determined.

### Subjects

Only original, quantitative, peer-reviewed papers were taken into account. Our search strategy was further narrowed by only addressing studies performed within western industrialized countries. Furthermore, only minority immigrant groups originating from non-industrialized countries were included. Non-industrialized countries were defined as all non-OECD member states (except Turkey and Mexico). Moreover, due to their specific situation, studies targeting at illegal immigrants, refugees, homeless people or handicapped people were not included. Also studies specifically addressing the primary medical care use of children or adolescents were not included. The majority population served as the non-immigrant reference group. Therefore, studies without an indigenous majority group were excluded. Non-immigrant minority groups like Afro-Americans in the United States (US) and American Indians in the US and Canada were also not included in the review.

### Outcome measures

For the purpose of our review, only studies concerning the actual use of primary medical care were included. Primary medical care was defined as the provision of accessible health care services by clinicians who are accountable for addressing a large majority of health care needs, developing a sustained partnership with patients and practicing in the context of the family and the community [18]. The relevance of studies for our review relied on such commonly recognized attributes of primary medical care as accessibility, comprehensiveness, first contact care, general scope, coordination, continuity and accountability [19-22]. This means that studies concerning family physician care, outpatient care, private surgery care and care from a primary health centre were included in our review. However, countries vary in the extent that primary medical care can be distinguished from secondary and tertiary care. In the Unites States emergency rooms function as first contact care especially for vulnerable groups. To enhance the comparability between countries, primary medical care was therefore operationalised as care provided by physicians with a specialty in family practice, general practice, general internal practice, obstetrics and gynaecology, outpatient specialist care or emergency room care for countries where a strong gate-keeping system is absent. The search strategy was narrowed by including only general health care use for physical problems. If it was clear that studies were aimed specifically at mental health problems, mental health care, care for specific diseases, palliative care, dental care and medication use, they were excluded.

### Search strategy

For this review we initially consulted PubMed, PsycInfo, Cinahl, Sociofile, Web of Science and Current Contents electronic databases for the period 1980 to May 2003. The search strategy was performed by a librarian and aimed at a high sensitivity, ensuring the inclusion of as many relevant papers as possible. The databases were searched using the MeSH terms formulated in PubMed (Appendix 1). For the sake of sensitivity the initial search was performed regardless of context of care. In addition, for the period May 2003 to January 2006 the results were updated by a comparable search in PubMed and PsycInfo only addressing primary medical care. No language restrictions were applied and no additional hand searches were performed. No authors were contacted for additional information. Where possible, additional information was retrieved from the Internet.

### Study selection

The titles of the papers were examined by four researchers (EU, WD, PG and MF), each title was screened by two researchers independently of each other to assess appropriateness for inclusion (answer categories yes, doubt, no). First appropriateness was judged based on the titles. A paper was excluded in case two researchers agreed that one or more of the above inclusion criteria were not met in the title. In all other cases abstracts were retrieved and again screened by two reviewers. A paper was included in the review when two reviewers felt that the abstracts revealed that all inclusion criteria were met. A paper was excluded if both reviewers decided that one or more criteria were not satisfied. Where no consensus between 2 reviewers was reached, a decision was made in a consensus meeting with two reviewers (EU and WD). All remaining papers were judged based on the full text according to a similar procedure.

### Quality assessment

In our review the study quality will be related to the likelihood of reporting significant differences in primary care use between immigrant and non-immigrant groups. Table 1 provides an overview of the quality indicators used in our review. These indicators are frequently used in quality assessment of observational studies [23-27]. The quality of the studies was assessed by 2 reviewers independently of each other (EU and WD). In case of disagreement, consensus was achieved in a meeting with two reviewers (EU and WD). The overall quality score was included in the analyses as a linear variable. In addition to the overall quality of the study, whether or not a culture-/language-adjusted questionnaire was used and whether the study adjusted for potential confounders was added as a separate variable in the multilevel analyses (0 = no, 1 = yes).

**Table 1:** Methodological quality assessment of studies included in the review (n = 37)

**Study population:**	
Were the groups clearly defined?	8 studies unclear/no 29 studies yes
Can selection bias sufficiently be excluded? ^1^	11 studies unclear/no 26 studies yes
Did the immigrant groups and the majority population originate from the same source population? ^2^	2 studies unclear/no 35 studies yes
	
**Measurement:**	
Was the data collection adjusted for possible language problems or cultural differences^3^	24 studies unclear/no 13 studies yes
Was use of primary medical care determined independently of immigrant status?^4^	28 studies unclear/no 9 studies yes
Was immigrant status determined independently of primary medical care use?^4^	14 studies unclear/no 23 studies yes
**Analysis:**	
Were the results adjusted for potential confounders?	11 studies unclear/no 26 studies yes

1 Selection bias was only expected to be sufficiently excluded when the study population was based on a random selection from a national sample.

2 Immigrant groups and the majority population originated from the same source population when both samples where retrieved from the same basic population

3 Adjustment for possible language problems and cultural differences was accomplished when during the data collection for instance interpreters or translated questionnaires were used.

4 The use of primary medical care was determined independently from immigrant status (and the other way around) when it was impossible that a person's score on the use of care could be influenced by knowledge about a person's immigrant status. This was not the case when a physician treating the patient filled in both the health care use and a person's immigrant status

### Analyses

If studies reported more than one different outcome measure for primary medical care, all measures were included in our review. Given the fact that outcome measures are nested within studies, the structure of the data is hierarchical. To account for this hierarchical structure, logistic multilevel analyses were performed to answer the research questions concerning the association between the likelihood of significant differences between immigrant and non-immigrant groups in the use of primary medical care and strength of the primary care system, study characteristics and quality of the study (using MLwiN) [28]. In each study and for each immigrant group the significance of differences in use with the non-immigrant group was determined. This way a dichotomous independent variable could be calculated (0 = no significant difference in use, 1 = significant difference in use). The individual studies were interpreted as the highest level, whereas outcome measures were defined at the lower level. If multiple results for the same outcome measure were presented, the most adjusted result was retrieved. Significant differences in use were determined at alpha = 0.05 level. Where significance level was not mentioned in the paper, if possible the significance of differences was calculated by using additional information presented in the paper. Significance was assumed in cases of very large sample size (> 150.000 persons included). In all other cases the significance of differences in use remained unclear.

Given the expected large variation in study characteristics, attention will also be paid to the association between study results and study characteristics. The following study characteristics were included in the logistic analyses: sample size for each migrant group, length of the measurement period of use, publication year, adjustment for confounders at the outcome level and commonly used confounders in the analyses. To explore if significant differences in primary care use varied across immigrant groups, this variable was reduced to four subgroups for power reasons. This reduction was based on distinguishing immigrant groups originating from the African, Asian, American and European continent. If studies did not specifically define the immigrant groups, a mixed category label was given. In most of the studies the mixed category label is referring to a subgroup within the study population, in addition to more specifically defined immigrant groups (see Additional file 1). The strength of the primary care system in the countries represented in our review was based on scores used in a study among OECD member states [14]. In this study the strength of the primary care system was calculated for each OECD country based on a wide range of primary care system characteristics like accessibility, longitudinality and community orientation. The distribution of the scores was very skewed, with a weak primary care system in the US and strong primary care systems in the European countries, represented in our review, and Canada. This resulted in a dichotomisation of countries (0 = other countries, 1 = United States). Our review was restricted to the adult population; however not all studies made a clear distinction between adults and children. If possible, only results from the adult population were included, otherwise the overall results were retrieved.

## Results

### Study descriptions

**Figure 1:**
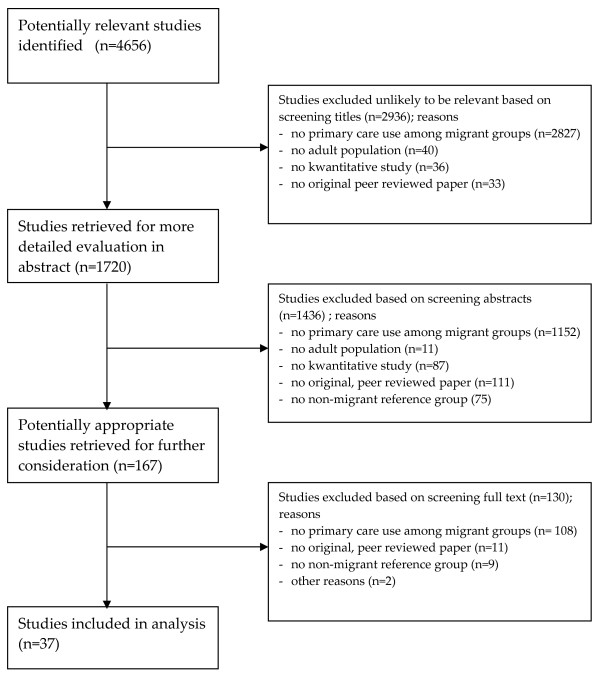
**Flow of included studies**. The figure shows the numbers of included and excluded studies during the review process.

The application of the search strategy to the specified databases resulted in 4,656 hits (4,404 from the initial search and 252 from the additional search). Based on the titles and abstracts 167 studies were selected which possibly met the inclusion criteria (figure 1). Based on the full text of the papers, it was concluded that 37 papers fulfilled all the inclusion criteria. Of these 37 papers 7 at least partly described the same datasets (see Additional file 1). As the outcome measures of these studies differed, all 7 were included in our review.

### Subjects

A wide variety of immigrant groups were included in the studies (see Additional file 1). Most attention was paid to Hispanics, Turkish and Asian groups. Not surprisingly this focus was strongly related to the host country as the studies were performed within 7 different countries. The definition of immigrant status was most often based on the person's country of birth (n = 6), country of birth of the parents (n = 2) or a combination of both (n = 1). In addition, self-identification was often applied (n = 5) sometimes combined with other measures like place of birth and most spoken language (n = 2). Less frequently name recognition (n = 1) or perception of the physician was used (1).

### Study findings

As some studies reported more than one outcome measure, in total 108 different outcome measures of primary medical care were included (see Additional file 1). Most often primary medical care was operationalised as family physician care (GP) (n = 42). Other outcome measures referred to outpatient specialist or emergency room care (n = 24) and a doctor's office or primary health care centre (11). When results were presented for different immigrant groups separately, outcome measures were derived for each immigrant group. This way it was possible to take immigrant groups as a variable in our review.

The original 108 overall outcome measures therefore resulted in 252 outcome measures for each immigrant group separately. The number of outcome measures varied from country to country. Overall, a significantly higher primary care use among immigrant groups as compared to the non-immigrant group was found in 20.2% of the outcome measures; 27.4% reported a lower use, 44.0% showed no significant differences and in 8.3% of the cases significance was unclear. Multilevel logistic regression analysis showed that studies performed within the US were more likely to find significantly different results than studies performed in the other countries (Table 2). Most often these significant differences in the US were in the direction of a lower use among immigrant groups (Table 3).

**Table 2:** Significant differences between immigrant an non-immigrant groups in use of primary care by quality aspects and study characteristics (Multilevel logistic regression, B and standard error)*

	B	Se
Intercept	0.23	0.16
		
Quality aspects:		
Total quality score	**0.75**	**0.19**
Adjustment for confounders at study level	**-2.34**	**0.81**
Culturally adjusted questionnaire	**-1.83**	**0.52**
		
Study characteristics:		
Country US	**1.67**	**0.60**
Publication year	-0.00	0.04
Adjustment confounders outcome level	-0.00	0.41
Sample size majority reference group	0.00	0.00
Sample size immigrant groups	-0.00	0.00
Length of reference period of use	-0.15	0.26
Background immigrant groups ^a^		
European	1.69	0.92
African	0.14	0.77
Asian	0.92	0.56
(South/central) American	0.23	0.60
		
Variance study level^b^	0	0
		
Variance outcome level	**0.92**	**0.09**

* significant differences are printed in bold (p < 0.05)

a the mixed immigrant category served as the reference group

b the introduction of variables at study level resulted in the disappearance of the initial variance at the study level compared to the 0 model with only a constant

**Table 3:** Differences between immigrant and non-immigrant groups in primary medical care by significantly related variables (%)

	Higher use	Lower use	No significant differences	Significance unclear
Adjustment for confounders at study level (%):				
yes	18.6	25.8	47.1	8.6
no	32.3	38.7	22.6	6.5
				
Culture/language adjusted questionnaire (%):				
yes	10.3	30.2	60.0	-
no	30.6	24.6	28.6	16.7
				
Adjustment for health status (%):				
yes	21.6	15.7	60.8	2.0
no	19.9	30.3	39.8	10.0
				
Country (%):				
US	10.1	55.1	32.6	2.2
other countries	25.8	12.3	50.3	11.7

In contrast to the country effect, the significance of differences in health care utilization was not dependent on the immigrant groups studied. Although a large variety of immigrant groups were studied (n = 25), no overall consistent patterns could be distinguished. This implies that the country and thus possibly the strength of the primary health care system is a stronger predictor of differences in use than the immigrant groups using care. The year of publication was not related to the significance of the differences found, suggesting that in general differences between immigrant and non-immigrant groups in primary care use did not change substantially over time. Moreover, the length of the reference period of use, adjustment for confounders at the outcome level, number of persons included with either a immigrant background or indigenous majority background did not change the results. Lack of power complicated the multilevel analyses exploring predictors of a higher or lower use among immigrant groups. However, the retrieved results confirmed the importance of the country factor (not shown).

### Methodological study quality

Overall, studies met 2 to 6 of the 7 quality indicators (Table 1). Most studies stated a clear definition of the immigrant groups (n = 29), excluded bias sufficiently (n = 26), adjusted at least some outcome measures for potential confounders (n = 26) and used the same source population for all immigrant groups (n = 35). One third of the studies took cultural differences and language problems during the data collection into account. Common means to handle cultural differences and language problems were the use of a bilingual interviewer or translated questionnaires. Logistic multi-level analysis emphasized the importance of taking into account cultural differences and language problems. Studies adjusting for cultural differences and language problems less frequently reported significant differences in use of primary medical care between immigrant and non-immigrant groups than studies not taking this into account. Studies not adjusting for potential language or cultural problems were more likely to report a relatively higher use among immigrant groups (Table 2 and 4). The same applied to studies including confounders in the analyses as these studies also less frequently found significant differences in primary care use between immigrant and non-immigrant groups. In addition, the direction of the differences was not comparable. Significant differences were more often in the direction of a higher use among immigrant groups in studies not adjusting for confounders, whereas studies taking confounders into account more often reported a lower use among immigrant groups. In-depth analyses showed that of the most frequently applied confounders (age, sex, education and health status), health status clearly related most strongly to differences between non-immigrant and immigrant groups in primary care use (not shown). Studies not adjusting for health status more frequently reported a lower use among immigrant groups compared to studies adjusting for health status (Table 3). Furthermore, the overall quality score of the studies was positively related to the likelihood of reporting significant differences. Higher quality scores increased the likelihood of significant differences (Table 2).

## Discussion

Research attention for differences between immigrant and non-immigrant groups in primary care use has increased over the years. Nevertheless, to our knowledge no systematic attention has been paid to the synthesis of results from the various studies. In our review, literature was systematically reviewed, resulting in the inclusion of 37 studies from 7 countries. With respect to the extent to which countries and immigrant groups differ in primary medical care use from the indigenous majority population, we conclude that no overall consistent pattern could be distinguished with respect to immigrant groups. Generally, immigrant groups do not make an excessive demand upon the primary care system nor do they opt out [29]. However, the significance of differences in use varied across countries. Compared to the other countries, studies performed in the US more often reported significant differences between immigrant groups and the majority population, especially in the direction of a lower use among immigrant groups. As the strength of the primary care system in the US is found to be substantially weaker than in the other countries, our results suggest a relationship between differences among immigrant and non-immigrant groups in use and a country's orientation towards primary care. Possibly a strong primary care system positively contributes to equity in access for potentially vulnerable groups. This issue clearly needs to be addressed in future research as other studies suggest that psychological and cultural characteristics (e.g. adherence to Asian values) in help seeking strategies explain differences in use of care more than health system related characteristics [30]. Other research underlined the relative importance of education and income for explaining differences in use between immigrant groups in contrast to health system related variables [31].

Study outcomes were found to be related to the quality indicators. In general a higher overall methodological quality score increased the chance of significant differences. Nevertheless, more detailed aspects of the study quality were inversely related to the likelihood of significant differences in primary care use. Studies allowing for potential language problems or cultural differences during the data collection and potential confounders in the analyses less frequently reported significant differences as compared to studies not adjusting specifically for these aspects. In addition, the direction of the differences was not comparable. For instance, studies not adjusting for health status more frequently reported a lower use among immigrant groups compared to studies adjusting for health status. Subsequently, the results from studies lacking the inclusion of confounders as health status and attention for cultural and language problems seem more inclined to report differences between immigrant and non-immigrant groups in health care use that are actually reflecting methodological shortcomings than existing differences between immigrant groups. For instance, neglecting possible cultural and language problems might result in a selective response of people from immigrant groups. Nevertheless, given the contrasting findings between specific quality aspects and the overall methodological study quality, this issue clearly needs more research attention. However, the importance of taking cultural differences and language problems into account is in line with research in this field suggesting that these factors affect the validity of self-reported data from immigrant groups [32-34]. The fact that confounders are clearly not equally divided across immigrant groups emphasizes the need for including confounders in the analyses concerning differences between immigrant and non-immigrant groups in health care use. Our results especially emphasized the importance of including health status in the analyses, which is consistent with other research stating that a higher use of health care among immigrant groups is often related to their poorer health status [3].

Our conclusions should be considered in the light of the following limitations. First, it has to be mentioned that non-significance in some studies might be due to a power problem instead of the absence of differences between immigrant and non-immigrant groups [35]. Nevertheless, our analyses were controlled for the sample size of the immigrant groups and this was not related to the chance of significant differences in use. Although lack of power might be an issue for some studies, this suggests no large power problem across the studies included in the review. Our review focused on primary care use for physical problems, excluding use for mental health problems. Presumably differences between immigrant and non-immigrant groups in utilization for mental health problems will show a different pattern, as research suggests that cultural factors possibly play a role in the reluctance to consult for psychosocial problems. Some immigrant groups are found to have a tendency to somatise psychosocial problems, which might in turn be an explanation for a higher primary care utilization [36]. Since health status proved to be a crucial measure in health care utilization studies, future research needs to consider possible cultural differences in self assessed health [37]. Another issue that possibly negatively influenced the results is the fact that in order to take into account the different country profiles of primary care we did not use the same definitions for all countries. This was particularly the case concerning emergency room care in the United States. It can be questioned whether ER care really reflects primary medical care. Given the fact that especially for vulnerable groups emergency room care in some countries shows characteristics of primary care, we decided to include this type of care when it was clear that it was not primarily emergency care for severe acute illness or accidents. Although most studies included a clear description of the immigrant groups, this classification varied largely, complicating the comparability of studies. The adequacy of immigrant background information collected in research has been discussed frequently [13]. Moreover, because we had to rely on the definitions and main categories applied in the individual studies, it was not possible to distinguish between ethnicity and immigration history (being a newcomer). As use of primary care presumable will be related to both aspects, this type of information would have provided useful information concerning the separate and combined effect of these aspects on the use of primary care.

The appropriateness of assignment to immigrant groups needs to be investigated and further developed. For power reasons in our analyses the various immigrant groups were reduced to four subgroups based on whether immigrants originated from the African, Asian, American and European continent. This reduction does not justify the large variation between immigrant groups from one continent, e.g. in case of immigrants from the Indian subcontinent and immigrants from South-East Asia. Furthermore, it is not evident that using the indigenous majority population's level of use provides a socially optimal benchmark [30]. It is possible that higher levels of use among the majority population represent over-utilization compared to their actual need. Moreover, it is not clear to what extent the differences between immigrant and non-immigrant groups observed are a result of differences among immigrant groups in individual preferences for health care which may or may not be reflective of problems with access to care [30]. For instance a possible preference for complementary or specialized care is not accounted for in the dependent variable of our review. Finally, the existence of significant differences in primary care use between immigrant groups is followed by the question addressing the exact extent of these differences. As our review focused on the likelihood of significant differences between immigrants groups, future research will need to address this issue more in detail.

## Conclusion

In conclusion, our review underlined the need for careful design in studies of differences between immigrant and non-immigrant groups in health care use. In general a higher overall methodological quality score increased the chance of significant differences. Our study suggests that, compared to the majority population, immigrant groups do not make an excessive demand upon the primary care system nor do they opt out. However, the significance of differences between immigrant and non-immigrant groups in use of primary care services varied across countries. Our review clearly showed that, compared to the other countries, studies performed in the US more often reported significant differences between immigrant groups and the majority population, in the direction of a lower use among immigrant groups. This might be interpreted as a reflection of a weaker primary care system in the US compared to Europe and Canada.

## Competing interests

The authors declare that they have no competing interests.

## Authors' contributions

The titles of the papers were examined by EU, WD, PG and MF to assess appropriateness for inclusion in the review. EU performed the search strategy, abstracted the data, assessed the quality of the studies, participated in the statistical analysis and drafted the manuscript. WD participated in the assessment of the quality of the studies. PS performed the statistical analysis and commented on the draft of the manuscript. WD, MF, PG provided valuable comments on the search strategy, data analysis and draft of the manuscript. Al authors read and approved the final manuscript.

## Appendix 1 MeSH terms used in the search strategy

[Health Services OR Hospitals OR Rehabilitation OR Residential Facilities

OR Primary Health Care OR Ambulatory Care Facilities OR Use Or Utilization

OR Utilization OR Patient Care OR Health services Accessibility

OR Health Services/utilization OR Ambulatory Care Facilities/Utilization

OR Hospitals/Utilization OR Rehabilitation/Utilization/Residential Facilities/Utilization

OR Primary Health Care/Utilization]

AND

[Transients and Migrants OR Migrant groups OR Minority Groups

OR Emigration and Immigration OR Cultural diversity OR Cross-cultural Comparison

OR Acculturation OR Cultural Characteristics OR Cultural Deprivation]

Limits: All Adults: 18+ years, Editorial, Review, Letter, Comment (publication type)

## Pre-publication history

The pre-publication history for this paper can be accessed here:



## Supplementary Material

Additional file 1 **Supplementary table: Studies included in the review by host country (n = 37)**. The table provides a detailed overview of characteristics of the included studies in the review. Click here for file
