# Supplementary material for: Differences between immigrant and non-immigrant groups in the use of primary medical care; a systematic review
Source: BMC Health Serv Res. 2009 May 11;9:76. doi: 10.1186/1472-6963-9-76 (PMC2689181; doi:10.1186/1472-6963-9-76)
Supplement: Additional file 1 — Supplementary table: Studies included in the review by host country (n = 37). The table provides a detailed overview of characteristics of the included studies in the review. [file 1472-6963-9-76-S1.doc]

| Authors and source | Sample | Immigrant groups | Outcome measure | Major findings |
| --- | --- | --- | --- | --- |
| United Kingdom |  |  |  |  |
| Livinston et al., 2002[1] | ≥65 years olds living in urban poverty area | African/Caribbean | GP use (3 months)  hospital outpatient services (3 months) | no significant difference ^a^  no significant difference ^a^ |
| Lindesay et al., 1997[2] | ≥65 years old living Leicester | Hindu Gujaratis | GP use (1 month) | higher in immigrant group ^a^ |
| Smaje et al., 1997[3]^, #^ | national general population | Chinese  Caribbean  Indian  Pakistan  Bangladesh  African  other | GP use among respondents reporting illness (2 weeks, responses from 8 years)  GP use among respondents reporting no illness (2 weeks, responses from 8 years)  outpatient services among respondents reporting illness (3 months, responses from 8 years)  outpatient services among respondents reporting no illness (3 months, responses from 8 years)  GP use among people aged 0-44 (2 weeks, responses from 8 years)  GP use among people aged >45 (2 weeks, responses from 8 years)  outpatient services among people aged  0-44 (3 months, responses from 8 years)  outpatient services among people aged >45 (3 months, responses from 8 years) | higher among Indian and Pakistan ^a^  no significant difference ^a^  no significant difference ^a^  lower among Indian, Pakistan, Chinese and the mixed population ^a^  lower among African and Chinese ^b^  higher among Indian and Caribbean ^b^  lower among Indian, Pakistan, Bangladesh, Caribbean and the mixed population ^b^  higher among Caribbean and African ^b^ |
|  |  |  |  |  |
| Balarajan et al., 1989[4]^, #^ | national population aged 0-64 | West Indian  Indian  Pakistan | GP consultation for men aged 16-64  (2 weeks)  GP consultation for females aged  16-64 (2 weeks) | higher for all immigrant groups ^b^  higher for Pakistan ^b^ |
| Ritch et al., 1996[5] | > 65 years old living in inner city wards Birmingham registered at a practice | Asian  West Indian | GP use (1 year) | no significant difference ^a^ |
| Johnson et al., 1983[6] | <60 years old inhabitants inner areas of the West Midlands conurbanation | Asian  Afro-Caribbean | no visit to the family physician (1 year)  1-2 visits to the general practitioner (1 year)  3-5 visits to the family physician (1 year)  ≥ 6 visits to the family physician (1 year)  visit to outpatient or emergency clinic while bypassing the family physician | lower among Asians ^a^  lower among Asians ^a *^  higher in immigrant groups ^a *^  higher among Asians ^a^  ^*^  lower among immigrant groups, significance unclear ^a^ |
| Liao et al., 1995[7] | Chinese inhabitants of greater Glasgow health board compared to the general Scottish population | Chinese | number of consultations with GP (1 year) | lower among immigrant group ^a^ |
| Murray et al., 1986 [8] | 16-64 years old adult population living in west London | Asian | GP use among men on own account  (2 weeks)  GP use among women on own account  (2 weeks) | higher among immigrant group ^b^  no significant difference ^b^ |
| Baker et al., 2002[9] | adult population from 10 GP practices in each of 6 health authorities | immigrants | GP consultation for condition-specific morbidity | significantly higher among immigrant groups for backache, indigestion, sleep problems, migraine, cold/flu ^b^ |
| Gillam et al., 1989[10] | patients registered at an urban group general practice in London | Asian  West Indian | GP consultation ratio for males (1 year, the total number of consultations observed divided by the total expected number)  GP consultation ratio for females (1 year)  number of male consulting GPs with a particular condition (1 year)  number of females consulting GPs with a particular condition (1 year) | higher among Asian, lower among West Indian ^c^  higher among Asian, lower among West Indian ^c^  lower among West Indian ^c^  lower among Asian and West Indian ^c^ |
| Canada |  |  |  |  |
| Blais et al., 1999[11] | non institutional population ≥15 years olds Quebec | members of immigrant groups | number of outpatient or emergency room care (1 year)  1-2  3-5  6 or more  number of contact with GP (1 year)  0  1-2  3-5  6 or more  number of private office visits  1-2  3-5  6 or more | no significant difference ^d^  no significant difference ^d^  no significant difference ^d^ |
|  |  |  |  |  |
| Wen et al., 1996[12] | non-institutionalized population province Ontario (16-64 years old) | Caribbean  Asian born in Canada  Asian immigrated >10 years  Asian immigrated  < 10 years  other ethnicity  other ethnicity immigrated >10 years  other ethnicity immigrated <10 years | GP use (1 year)  emergency room use (1 year) | lower among Asian born in Canada, higher among Asian and other immigrant groups immigrated > 10 years and other immigrant groups immigrated <10 years ^b^  lower among Asian immigrated <10 years and other ethnicity immigrated > 10 years ^b^ |
| United States |  |  |  |  |
| Langwell et al., 2002[13] | enrollees Medicare | Asian  Hispanic/Latino | no visits to doctor’s office (6 months)  5 or more visits to doctor’s office  (6 months)  any emergency room use (6 months) | higher among both immigrant groups ^a^  lower among Asians ^a^  higher among Hispanic/Latinos and lower among Asians ^a^ |
| Ku et al., 2001[14] | general population aged <65 years old | Hispanic  Asian | any emergency room use (1 year)  number of emergency room contacts (1 year) | no significant differences ^b^  no significant differences ^b^ |
| Weinick et al., 2000[15]^, #^ | national non-institutionalized population | Hispanics | any ambulatory physician visit (most recent measure from 3 measurements during 20 years)  mean number of ambulatory physician contact (most recent measure from 3 measurements during 20 years) | lower among immigrant group ^b^  lower among immigrant group ^a *^ |
|  |  |  |  |  |
| Schur et al., 1987[16]­­^, #^ | national general population | Puerto Rican  Mexican  Cuban  Other Latin  Other Hispanic | ambulatory physician visit during (1 year)  number of physician visits during (1 year) | Puerto-Ricans slightly higher and other groups (some slightly) lower use, significance assumed ^a^  other Latinos higher and all other groups lower, significance assumed ^a^ |
| Cornelius, 1993[17]^, #^ | national general population | Hispanics  Asian | ambulatory physician visit (1 year) overall  among people in fair/poor health  among people in fair/poor health and private insured  among people in fair/poor health and public insured  among people in fair/poor health and no insurance  number of ambulatory physician visits (1 year)  overall  among people in fair/poor health  among people in fair/poor health and private insured  among people in fair/poor health and  public insured  among people in fair/poor health and no  insurance | lower among immigrant groups, significance assumed ^a^  lower use among immigrant groups, significance assumed ^b^ |
| Pourat et al., 2000[18] | ≥ 65 years old living Los Angeles | Korean | number of ambulatory doctor’s office visits past year | higher in immigrant group ^b^ |
| Yu et al., 1982 [19] | national ambu-latory patients to non-federal employed physicians prin-cipally engaged in office practice | Asian-Pacific | office-based physician visit in a randomly assigned weekly reporting period aged groups  15-24  25-44  45-64  ≥65 | In all age groups lower use among immigrant group, significance assumed ^a^ |
|  |  |  |  |  |
| Guendelman et al., 2000[20] | national adult population | Latinos | non-emergency outpatient room care  (1 year)  emergency room care (1 year) | no significant difference ^b^    no significant difference ^b^ |
| Wells et al., 1988[21] | general population Los Angeles | Mexican | any outpatient visit for physical problems (6 months) | lower use among immigrant group ^b^ |
| Washington et al., 2002[22] | national adult veterans | Asian-Pacific  Hispanic | any ambulatory Veterans Affairs (VA) health care use (1 year)  VA-only ambulatory care use (1 year)  dual VA/non-VA ambulatory care (1 year)  non-VA-only ambulatory care use (1 year)  number of VA-only ambulatory care visits (1 year)  number of dual VA/non-VA ambulatory care visits (1 year)  number of non-VA-only ambulatory care visits (1 year)  overall number of ambulatory care visits (1 year) | higher among Hispanics ^b^  higher among Hispanics ^a^  higher among Hispanics ^a *^  lower among Hispanic ^a *^  higher among Asian-Pacific ^a^  no significant difference ^a^  no significant difference ^a^  significance is unclear ^a^ |
| Zuvekas et al., 2003[23]^, #^ | national general non-institutionalised population | Hispanic | any non-emergency room ambulatory treatment from office based providers and outpatient departments of hospitals (most recent measurement from 3 measurements over a 3-year period)  number of visits to these providers (most recent measurement from 3 measurements over a 3-year period) | lower in immigrant group ^a^  lower in immigrant group ^a^ |
| Weinick et al., 2004[24]^, #^ | national general population | Asian  South American  Mexican  Cuban  Puerto Rican  Central American and Caribbean | any ambulatory health care use (1 year)  any emergency room use (1 year) | lower among all groups ^b^, except for Puerto Rican and South American not significant  lower for Asian, Mexican and Cuban ^b^ |
| Hargraves et al., 2001[25] | national no elderly (<66 years old) with a public or private health insurance | Hispanic | proportion of physician visits in ER during past year  proportion of physician visits in ER during past year among persons in plans without gate keeping  proportion of physician visits in ER during past year with gate keeping | higher among immigrant group ^a^  no significant difference ^b^  no significant difference ^b^ |
| Baxter et al., 2001[26] | older (>60 years old) residents of a rural area in Colorado | Hispanic | number of outpatient visits (to clinic and emergency rooms)(1 year) | no significant difference ^b^ |
| Andersen et al., 1986[27] | national general population | Hispanic | number of medical doctors visits (1 year)  any physician visits (1 year)  proportion outpatient department/ emergency room visits of total physician visits (1 year) | no significant difference ^a^  no significant difference ^a^  no significant difference ^a^ |
| Rosenbach et al., 1995[28] | non-institutionalised national Medicare beneficiaries | Hispanic | outpatient department visit  emergency room care (person year) | no significant difference ^b^  no significant difference ^b^ |
|  |  |  |  |  |
| *Sweden* |  |  |  |  |
| Tomson et al., 1988[29] | adult population 4 health care centres Stockholm | Turks | visit primary health care centre (6 months) | higher among Turks, significance is unclear ^c^ |
| Hjern et al., 2001[30] | national adult population | Turks  Chileans  Iranians | emergency room care (3 months) | higher in all immigrant groups ^a *^ |
| Norway |  |  |  |  |
| Naess, 1992[31] | general population inhabitants part Oslo | ethnic minorities | office visit primary healthcare centre (10 months)  number of contacts primary health care centre | higher among minority groups ^a^  no significant difference ^a^ |
| Denmark |  |  |  |  |
| Norredam et al., 2004[32] | adult citizen residing in the catchments area of a specific hospital in Copenhagen | Pakistan  Iraq  Somalia  Turkey | number of ER contacts (1 year) | higher Turks and Somalians ^b^ |
| The Netherlands |  |  |  |  |
| Stronks et al., 2001 [33]^, #^ | adult population Amsterdam | Turks  Antilleans  Moroccans  Surinamese | any GP use ( 2 months) | higher among Moroccans ^b^ |
| Reijneveld, 1998[34]^, #^ | adult population Amsterdam | Turks  Antilleans  Surinamese  Moroccans  Other non-industrialized | GP use (2 months)  GP use past 2 months among 16-34 years old | higher among all immigrant groups except Antilleans ^b, *^  no significant difference ^b^ |
|  |  |  | GP use past 2 months among 35-64 years old | no significant difference |
| Kocken et al., 1994[35] | adult (16-75 years old) population city of Rotterdam | Surinamese | overall contact with GP ( 2 months)  contact with GP (2 months)  55-75  45-54  35-44  25-34  16-24 | higher in immigrant group ^b^  higher in immigrant group ^b^  higher in immigrant group ^b^  higher in immigrant group ^b^  no significant difference ^b^  no significant difference ^b^ |
| Weide et al., 1998[36] | national adult (18-64 years old) population registered at a GP practice | Turks  Surinamese  Moroccans | number of GP contacts per 1000 registered patients (3 months)  number of office contacts with GP among users | higher among Surinamese and Turks ^b^  higher for Turks and Surinamese ^b^ |
| Belleman, 1986[37] | adult population Amsterdam | Turks  Surinamese/Antillean  Moroccan  mixed | number of GP contacts past year  male 15-24  female 15-24  male 25-44  female 25-44 | higher in all immigrant groups, significance unclear ^a^  higher for all groups except among Moroccans significance unclear ^a^  higher in all groups, significance unclear ^a^  higher in all groups, significance unclear ^a^ |

# (partly) same data source

* significance is computed on basis data in paper, significance is not given in paper

a not adjusted for confounders

b adjusted for confounders

c standardized for age

d respondents were individually matched

Reference List

1. Livingston G, Leavey G, Kitchen G, Manela M, Sembhi S, Katona C: **Accessibility of health and social services to immigrant elders: the Islington Study.** *Br J Psychiatry* 2002, **180:**369-373.

2. Lindesay J, Jagger C, Hibbett MJ, Peet SM, Moledina F: **Knowledge, uptake and availability of health and social services among Asian Gujarati and white elderly persons.** *Ethn Health* 1997, **2:**59-69.

3. Smaje C, Grand JL: **Ethnicity, equity and the use of health services in the British NHS.** *Soc Sci Med* 1997, **45:**485-496.

4. Balarajan R, YUEN P, RALEIGH VS: **Ethnic differences in general practitioner consultations.** *British Medical Journal* 1989, **299:**958-960.

5. Ritch AE, Ehtisham M, Guthrie S, Talbot JM, Luck M, Tinsley RN: **Ethnic influence on health and dependency of elderly inner city residents.** *J R Coll Physicians Lond* 1996, **30:**215-220.

6. Johnson MR, Cross M, Cardew SA: **Inner-city residents, ethnic minorities and primary health care.** *Postgrad Med J* 1983, **59:**664-667.

7. Liao XH, McIlwaine G: **The health status and health needs of Chinese population in Glasgow.** *Scott Med J* 1995, **40:**77-80.

8. Murray J, Williams P: **Self-reported illness and general practice consultations in Asian-born and British-born residents of West London.** *Social Psychiatry* 1986, **21:**139-145.

9. Baker D, Mead N, Campbell S: **Inequalities in morbidity and consulting behaviour for socially vulnerable groups.** *British Journal of General Practice* 2002, **52:**124-130, lit.

10. Gillam SJ, Jarman B, Law R, White P: **Ethnic differences in consultation rates in urban general practice.** *British Medical Journal* 1989, **299:**953-957.

11. Blais R, Maiga A: **Do ethnic groups use health services like the majority of the population? A study from Quebec, Canada.** *Soc Sci Med* 1999, **48:**1237-1245.

12. Wen SW, Goel V, Williams JI: **Utilization of health care services by immigrants and other ethnic/cultural groups in Ontario.** *Ethn Health* 1996, **1:**99-109.

13. Langwell KM, Moser JW: **Strategies for Medicare health plans serving racial and ethnic minorities.** *Health Care Financ Rev* 2002, **23:**131-147.

14. Ku L, Matani S: **Left out: immigrants' access to health care and insurance.** *Health Aff (Millwood )* 2001, **20:**247-256.

15. Weinick RM, Zuvekas SH, Cohen JW: **Racial and ethnic differences in access to and use of health care services, 1977 to 1996.** *Med Care Res Rev* 2000, **57 Suppl 1:**36-54.

16. Schur CL, Bernstein AB, Berk ML: **The importance of distinguishing Hispanic subpopulations in the use of medical care.** *Med Care* 1987, **25:**627-641.

17. Cornelius LJ: **Ethnic minorities and access to medical care: where do they stand?** *J Assoc Acad Minor Phys* 1993, **4:**16-25.

18. Pourat N, Lubben J, Yu H, Wallace S: **Perceptions of health and use of ambulatory care: differences between Korean and White elderly.** *J Aging Health* 2000, **12:**112-134.

19. Yu ES, Cypress BK: **Visits to physicians by Asian/Pacific Americans.** *Med Care* 1982, **20:**809-820.

20. Guendelman S, Wagner TH: **Health services utilization among Latinos and white non-Latinos: results from a national survey.** *J Health Care Poor Underserved* 2000, **11:**179-194.

21. Wells KB, Golding JM, Hough RL, Burnam MA, Karno M: **Factors affecting the probability of use of general and medical health and social/community services for Mexican Americans and non-Hispanic whites.** *Med Care* 1988, **26:**441-452.

22. Washington DL, Harada ND, Villa VM, Damron-Rodriguez J, Dhanani S, Shon H, Makinodan T: **Racial variations in Department of Veterans Affairs ambulatory care use and unmet health care needs.** *Military Medicine* 2002, **167:**235-241.

23. Zuvekas SH, Tallaferro GS: **Pathways to access: Health insurance, the health care delivery system, and racial/ethnic disparities, 1996-1999.** *Health Affairs* 2003, **22:**139-153.

24. Weinick RM, Jacobs EA, Stone LC, Ortega AN, Burstin H: **Hispanic healthcare disparities: challenging the myth of a monolithic Hispanic population.** *Med Care* 2004, **42:**313-320.

25. Hargraves JL, Cunningham PJ, Hughes RG: **Racial and ethnic differences in access to medical care in managed care plans.** *Health Services Research* 2001, **36:**853-868.

26. Baxter J, Bryant LL, Scarbro S, Shetterly SM: **Patterns of rural Hispanic and non-Hispanic white health care use - The San Luis Valley Health and Aging Study.** *Research on Aging* 2001, **23:**37-60.

27. Andersen RM, Giachello AL, Aday LA: **Access of Hispanics to health care and cuts in services: a state-of-the-art overview.** *Public Health Rep* 1986, **101:**238-252.

28. Rosenbach ML, Adamache KW, Khandker RK: **Variation in medicare access and satisfaction by health status: 1991-93.** *Health Care Financ Rev* 1995, **17:**29-49.

29. Tomson Y, Lichtenstein P: **[Immigrants consult health care centers more often than Swedes].** *Lakartidningen* 1988, **85:**532-537.

30. Hjern A, Haglund B, Persson G, Rosen M: **Is there equity in access to health services for ethnic minorities in Sweden?** *Eur J Public Health* 2001, **11:**147-152.

31. Naess MH: **[Norwegian and immigrant patients at a health care center. Are there real differences?].** *Tidsskr Nor Laegeforen* 1992, **112:**361-364.

32. Norredam M, Krasnik A, Moller ST, Keiding N, Joost MJ, Sonne NA: **Emergency room utilization in Copenhagen: a comparison of immigrant groups and Danish-born residents.** *Scand J Public Health* 2004, **32:**53-59.

33. Stronks K, Ravelli AC, Reijneveld SA: **Immigrants in the Netherlands: equal access for equal needs?** *J Epidemiol Community Health* 2001, **55:**701-707.

34. Reijneveld SA: **Reported health, lifestyles and use of health care of first generation immigrants in the Netherlands: do socioeconomic factors explain their adverse position?** *Journal of Epidemiology & Community Health* 1998, **52:**298-304.

35. Kocken PL, Mackenbach JP, Oers JAMv, Uniken Venema HPH: **[Mortality, perceived health, self-reported use of health care services among Surinamese in Rotterdam].** *Tijdschrift voor Sociale Gezondheidszorg* 1994, **72:**231-236.

36. WEIDE MG, Foets M: **[Contacts of migrants in the Dutch general practice].** *Huisarts en Wetenschap* 1998, **41:**179-183.

37. Belleman SJM: **[Contactfrequency of minority patients].** *Huisarts en Wetenschap* 1986, **29:**48-50.
